# Severe COVID-19 May Impact Hepatic Fibrosis /Hepatic Stellate Cells Activation as Indicated by a Pathway and Population Genetic Study

**DOI:** 10.3390/genes14010022

**Published:** 2022-12-22

**Authors:** Leire Moya, Samaneh Farashi, Prashanth Suravajhala, Panchadsaram Janaththani, Jyotsna Batra

**Affiliations:** 1 School of Biomedical Sciences, Faculty of Health, Queensland University of Technology, Brisbane, QLD 4059, Australia; 2 Translational Research Institute, Queensland University of Technology, Brisbane, QLD 4102, Australia; 3 Amrita School of Biotechnology, Amrita Vishwavidyapeetham, Clappana, Kollam 690525, India; 4 Centre for Genomics and Personalised Medicine, Queensland University of Technology, Brisbane, QLD 4059, Australia

**Keywords:** COVID-19, in silico, risk variant, population studies, hepatic fibrosis/hepatic cell stellate pathway

## Abstract

Coronavirus disease 19 (COVID-19) has affected over 112 million people and killed more than 2.5 million worldwide. When the pandemic was declared, Spain and Italy accounted for 29% of the total COVID-19 related deaths in Europe, while most infected patients did not present severe illness. We hypothesised that shared genomic characteristics, distinct from the rest of Europe, could be a contributor factor to a poor prognosis in these two populations. To identify pathways related to COVID-19 severity, we shortlisted 437 candidate genes associated with host viral intake and immune evasion from SARS-like viruses. From these, 21 were associated specifically with clinically aggressive COVID-19. To determine the potential mechanism of viral infections, we performed signalling pathway analysis with either the full list (n = 437) or the subset group (n = 21) of genes. Four pathways were significantly associated with the full gene list (*Caveolar-mediated Endocytosis* and the *MSP-RON Signalling*) or with the aggressive gene list (*Hepatic Fibrosis/Hepatic Stellate Cell (HSC) Activation* and the *Communication between Innate and Adaptive Immune Cells*). Single nucleotide polymorphisms (SNPs) from the ±1 Mb window of all genes related to these four pathways were retrieved from the dbSNP database. We then performed Principal Component analysis for these SNPs in individuals from the 1000 Genomes of European ancestry. Only the *Hepatic Fibrosis/HSC Activation* pathway showed population-specific segregation. The Spanish and Italian populations clustered together and away from the rest of the European ancestries, with the first segregating further from the rest. Additional in silico analysis identified potential genetic markers and clinically actionable therapeutic targets in this pathway, that may explain the severe disease.

## 1. Introduction

Pandemics and deadly endemics have become more frequent in past decades [1,2,3,4,5]. COVID-19 is an infectious disease caused by the severe acute respiratory syndrome coronavirus 2 (SARS-CoV-2). It was first identified in China in December 2019 [6], and it has spread to 192 countries [7]. In Europe alone, nearly 26 million infected people and over 650 thousand killed have been reported as of February 2021 [7]. Not long after the beginning of the pandemic, the scientific community started to report relevant data related to this novel virus at the epidemiological and clinical levels [8,9,10,11,12,13,14,15,16,17,18,19,20], but it was unclear why some patients developed very severe diseases while most presented mild symptoms.

Ancestry studies have previously shown some populations are more susceptible to certain diseases [21,22,23]. Germline mutations have been associated with an increased risk of SARS-CoV-1 infection [24]. Furthermore, some populations initially appeared to be more severely hit than others with higher COVID-19-related deaths when the virus strains had not significantly evolved and were not divergent in different parts of the world, providing a platform to discover distinct genetic features in these populations. This was the case within the Spanish and Italian populations, which together accounted for nearly a third of the COVID-19 related deaths in Europe [25] and in spite of both countries implementing the first hard lockdowns in Europe [26]. Despite face masks and different restrictions of movement mandatory in Spain and Italy for many months after the beginning of the pandemic [27,28], and with high vaccination rates (~70%), both countries remained in the top ten European countries with the highest COVID-19 fatality rates accumulated 18 months later [25]. Some reports also suggest the initial death rate was underestimated and may have been 50% higher in Italy [29].

In this context, we hypothesised a list of candidate genes associated with SARS-like infections would build a valuable platform to which multiple in silico tools could be applied using the readily available genomic data from the European populations. Our analysis led to the identification of hepatic fibrosis/ hepatic stellate cells activation as the key pathway associated with the aggressive COVID-19, as indicated by the fact that Spanish and Italian populations clustered together and away from the rest of European ancestries for the SNPs in the genes related to this pathway. Our additional analysis shortlisted *IFNAR1* and *SERPINE1* as key genes influencing this pathway.

## 2. Methods

### 2.1. Identification of Candidate Genes for Analysis

PubMed was accessed between the 31st of March and the 25th of May 2020. The aim was to consolidate all empirically and predicted human genes reported to have a role during SARS-CoV-2 infection to date. This included those human genes that assisted with the viral entry, evasion of the host’s immune system and the SARS-CoV-2—human interactome (Figure S1). Those studies whose results did not include human genes interacting directly or indirectly with SARS-CoV-1 or SARS-CoV-2 or being affected by the disease they caused were excluded. After completing the literature research, all the genes identified were curated for the subsequent pathway and PC analyses. For example, when the literature reference did not specify the gene isoform (e.g., NF-κB), all those available (e.g., NFKB1 and NFKB2) were included in the analysis.

### 2.2. Canonical Signalling Pathway Analysis, Protein Interactions and GO Enrichment Pathway Analysis

Signalling pathway analysis was conducted using Ingenuity Pathway Analysis (IPA, QIAGEN) [30]. Gene symbols were entered for the Core Analysis Expression, with the *Ingenuity Knowledge Base* as reference. The top 20 signalling pathways were checked for gene overlap. For an easier visualisation, only those pathways with a minimum of five common genes were selected.

Those 21 up-regulated genes reported in COVID-19 severe patients were entered in the open database STRING (http://string.embl.de/ Last accessed on 20 June 2020) [31]. Data from Biocarta, BioCyc, GO, KEGG and Reactome databases are regularly curated and updated in this collection, which includes both physical and functional (non-direct) protein interactions. Our settings for the analysis sourced data from genomic context predictions, high-throughput assays, co-expression (conserved) and knowledge from existing databases. The default medium confidence score (0.4) was entered. The network was “zoom out” by allowing STRING to add immediate interactions. The network was clustered following the Markov Cluster Algorithm (MCL) clustering algorithms, as recommended by STRING’s users’ documentation [32].

GO analysis was done using Cytoscape plugin ClueGO [33]. This platform connects GO terms with pathway annotation networks such as KEGG, Biocarta, Reactome and WikiPathways. The 21 genes associated with severe COVID-19 phenotype were uploaded to generate the networks that reflect non-redundant relationships with the genes. The additional plugin CluePedia [34] allows visualising for interactions between the enriched pathways from reported experimental data. The regular and automated update of the networks makes this tool an up-to-date resource for the category analysed. The Kappa score was set to 0.5 and the functional enrichment analysis was based on the cut-off value of *p*-value < 0.05, with a hierarchical layout. All genes in the shortlisted signalling pathways were considered for further analysis.

### 2.3. Mortality Rate Frequency Calculation

The *cause-specific death rate* was measured using the formula provided by the Centers for Disease Control and Prevention [35], and the data are presented per 100,000 individuals. The total *COVID-19 associated deaths*, defined by the WHO International Guidelines (based on ICD) [36], were outsourced from the daily updated Coronavirus disease (COVID-2019) situation reports [25]. Every country’s population size was obtained from the Eurostat Data Browser [37] as of the 1st of January 2020. Public health data from the European populations studied here was searched for the most common COVID-19 comorbidities reported. Aging has been broadly identified as the main one [38,39,40], followed by cardiovascular disease (CVD), diabetes, chronic obstructive pulmonary disease (COPD) and cancer history [38,41]. Liver dysfunction, smoking status, chronic kidney diseases and immunodeficiency have also been reported [8,41,42].

### 2.4. Principal Component (PC) Analysis of Genomic Variants in Genes Identified by Pathway Analysis in European Populations

Genomic locations were annotated for all the genes from UCSC, GRCh37/hg19 built (genome.ucsc.edu/index Last accessed on 20 June 2020), and four lists were generated to include SNPs from the flanking regions of ± 1 Mb of the genes. Duplicates were removed, while all the transcript variants for any given gene were included for the selection of SNP and PC analysis, if available.

The genome-wide germline data of 2506 individuals were obtained from the 1000 genomes phase III v.5 b project, the latest release of the data (May 2013) (https://www.internationalgenome.org/ Last accessed on 20 June 2020). Starting from the genotype file vcf.gz formats provided by the 1000 genomes projects, standard quality control methods using PLINK v1.9 b (http://pngu.mgh.harvard.edu/purcell/plink/ Last accessed on 20 June 2020) [43] were performed to remove individuals with more than 3% missing genotypes, SNPs with a call rate < 97%.

PC analysis was conducted for the samples to determine population stratification. PCs were computed using PLINK software v1.9b for all samples of the 1000 Genomes (1 KG) project, phase 3 version 5b, to infer the ancestry of the samples based on the whole genome data. PLINK [43] was used to extract subsets of regions for a 1 Mb window of genes of our interest. PC1 and PC20 values > 6 standard deviations from five European ancestries; Great Britain (England and Scotland), CEU (Utah Residents with Northern and Western European Ancestry), Tuscany (Italy), Iberian (Spanish population) and Finland participants were used in the analysis.

### 2.5. Differential Allele Frequencies Analysis and Functional Annotations of SNPs

The allele frequencies were retrieved using the –freq command in PLINK software (as described above). A standard case/control association analysis using Fisher′s exact test was performed. In this analysis, Spanish and Italian samples were defined as cases, and other European samples were defined as controls (dummy variables). Consequent SNP pruning was performed with PLINK to calculate the LD between each pair of SNPs in the ±1 Mb window of the selected genes and to remove one of a pair of SNPs when the LD was high (r2 > 0.9). Next, in silico functional annotations of those *P*-significant variants were studied to identify the possible functional variants in those regions that were highly significant between cases (Spanish/Italian) and controls (the rest of Europe) and a RegulomeDB probability score was given [44]. This score ranges from 0 to 1, with 1 being the most likely to represent a regulatory variant, including those in non-coding regions [44].

## 3. Results

### 3.1. Shortlisted Candidate Genes and Their Role during Viral Infection

In May 2020, a total of 291 studies were shortlisted from an extensive literature search by entering the search terms shown in Figure S1. After removing duplicates, 95 abstracts were screened, and 50 studies were excluded due to falling the inclusion criteria. Finally, 45 publications were included to generate a candidate gene list for further pathway and genetic analysis.

From our first search, we identified 84 genes to have a role during SARS-CoV-1 and SARS-CoV-2 infections (Table 1 and Table 2) and 21 to be associated with COVID-19 aggressiveness (Table 3). In addition, 332 genes identified in the SARS-CoV-2–human interactome [11] were included. The remaining genes, reported in one study only, can be found in Table S1.

### 3.2. Signalling Pathways Involved in SARS Infection

The list of the above-identified genes was curated for the subsequent signalling pathway analysis. The two most significant canonical pathways associated with these genes were the *Caveolar-mediated Endocytosis* and *MSP-RON Signalling* pathways (*p* = 2 × 10^−19^ and *p* = 6.1 × 10^−19^, respectively). The full report of the analysis is listed in Table S2.

The *Caveolar-mediated Endocytosis* signalling pathway (Figure S2) controls different cellular processes such as endocytosis, cellular signalling and lipid recycling, which regulates the internalisation of different particles, including virus and bacteria [66]. The *MSP-RON Signalling* pathway (Figure S3) contributes to the macrophage-induced immune response, to assist the host in the viral recognition via the Macrophage Stimulating Protein (MSP) and the transmembrane receptor kinase RON Protein Tyrosine Kinase/Receptor [67].

### 3.3. Protein Interactions and Signalling Pathways Associated with COVID-19 Aggressiveness

**Figure 1:**
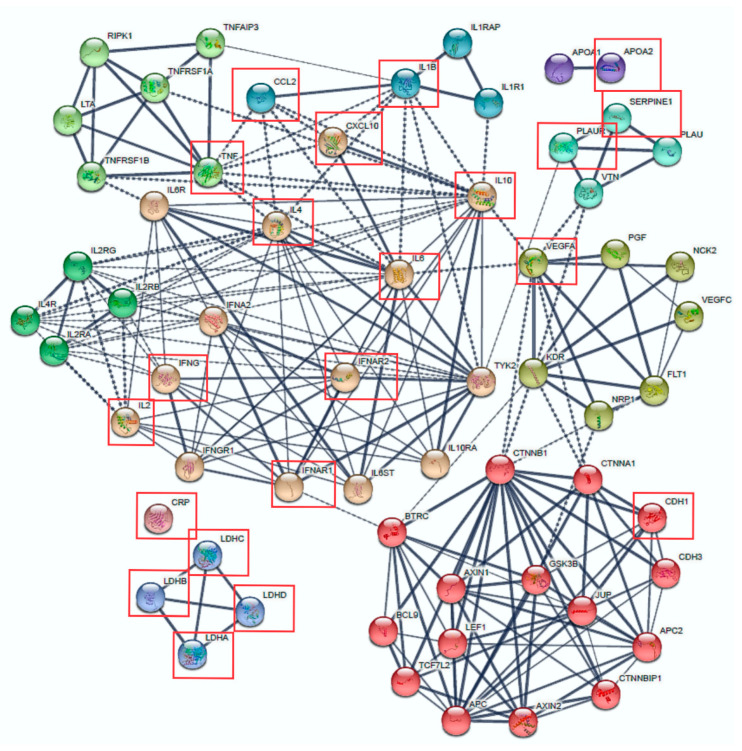
Protein–Protein Interaction network built from 21 candidate genes associated with COVID-19 aggressiveness. The 21 genes were entered in STRING (string-db.org/, accessed on 20 June 2020) and the network was enlarged to determine inter-cluster connections (dashed lines). The thickness of the lines is determined by the strength of the data support. Clusters were generated with the MCL algorithm with an inflation parameter of 3. Red squares mark the 21 backbone genes associated with COVID-19 severity.

Of the above shortlisted 84 genes, 21 coding genes were identified to be specifically associated with COVID-19 severe disease (Table S3). Figure 1 shows nine clusters of proteins and their inter- and intra-protein interactions with these 21 genes. Seven clusters had strong connections (based on STRING’s sourced data), as shown by the thickness of the lines. Three clusters remained independent (APO1/APO2 and LDH genes) and CRP did not cluster. Some proteins showed high connectivity with other clusters, such as IL4R, IL2RA, IL2RG, IL2RB, TNF, CCL2, IL4, IFNA2, IL1B, IL10, TYK2 and VEGFA, placing them as potential therapeutic targets to reduce the cytokine storm seen in severe COVID-19.

**Figure 2:**
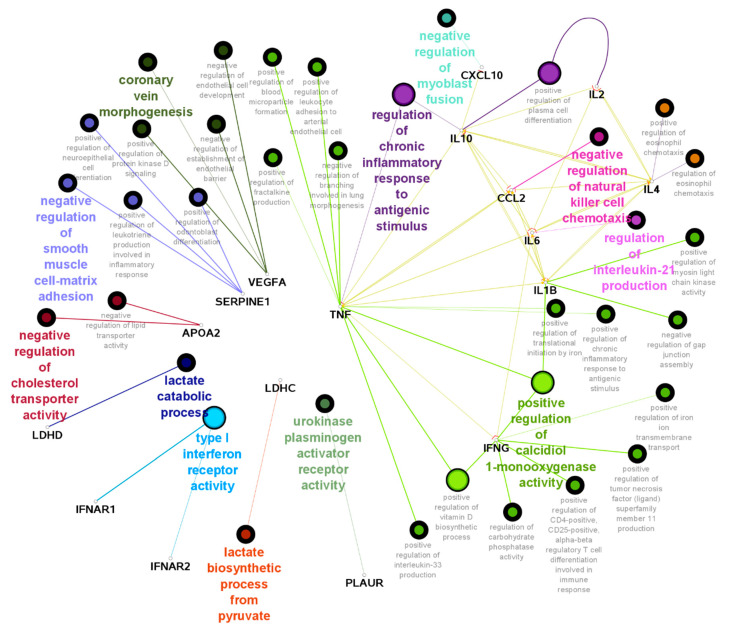
Enriched pathway analysis associated with COVID-19 aggressive-related genes.

GO analysis with these 21 genes is shown in Figure 2. Here, certain pathways show higher intra-connectivity, such as “regulation of chronic inflammatory response to antigenic stimulus”, “negative regulation of natural killer cell chemotaxis” and “regulation of IL-21 production”. These results correlate with the initial observations in COVID-19 severe patients, where there was an overproduction of cytokines/interleukins and a subsequent immune overreaction to the pathogen.

Signalling pathway analysis identified the *Hepatic Fibrosis/Hepatic Stellate Cell (HSC) Activation* and the *Communication between Innate and Adaptive Immune Cells* (Figure S4) as the two most significant signalling pathways (*p* = 2.5 × 10^−18^, *p* = 1.6.1 × 10^−14^ respectively). The activation of HSCs was the pathway representing the highest number of genes reported in severe SARS-CoV-2 infections (highlighted in purple in Figure S4). These include *IL6, IL1β, TNF-α, IL10,* and *IFN-γ* and *VEGF*, *MCP-1* as up- or down-stream regulators, respectively. The *Communication between Innate and Adaptive Immune Cells* (Figure S5) is the process in which both the immune and adaptive responses interact with each other to defend the host from infection [68].

### 3.4. Mortality Rate and Common COVID-19 Comorbidities Data in European Populations

Calculations of the mortality rates were done for the 27 countries that form the European Union (EU) [69]. This was first calculated on the 7th of April 2020 and updated later (26th of February 2021, Table S5). The UK was also included for its relevance to this study. To illustrate if the most reported COVID-19 comorbidities at the beginning of the pandemic (aging, heart disease, diabetes, smoking and liver disease) [8,38,41,42] were demographically homogeneous in Europe, we investigated the latest European reports available and where Spain and Italy appear on these reports. While Italy has the oldest population, Spain ranks 20th, with another 16 European countries in between [70]. Heart disease, the second most common COVID-19 comorbidity [39,64], is comparable between Northern and Southern European populations [71]. Next, we checked the status of diabetes. Both types I and II diabetes are slightly higher in North than South Europe [72,73]. Smoking habits studies done in European countries [74] place Spain in 4th place of incidence (combined current and ex-smokers, 52.2%), while Italy is the last one (30.2%).

### 3.5. PC Analysis Reveals Genetic Variants in Hepatic Fibrosis/HSC Activation Pathway Segregates Differently in European Populations

All SNPs located within ± 1 Mb flanking regions of the genes related to the top four pathways identified above—Caveolar-mediated Endocytosis MS*P-*RON Signalling Hepatic Fibrosis/ Hepatic Stellate Cell Activation and Communication between Innate and Adaptive Immune cells—were retrieved (Tables S6–S9). To investigate the minor allele frequency (MAF) of the SNPs located within these genomic regions, the allele frequencies of 1,121,451 variants were tested in European samples of the 1000 Genome study. A total of 81,271,745 genetic variants from 2504 people were included in the analysis after QC for the regions of interest. The minor allele of around 10% of these SNPs (before pruning) presented significant differences in Spanish/Italian populations when compared to other European populations (Table S10).

**Figure 3:**
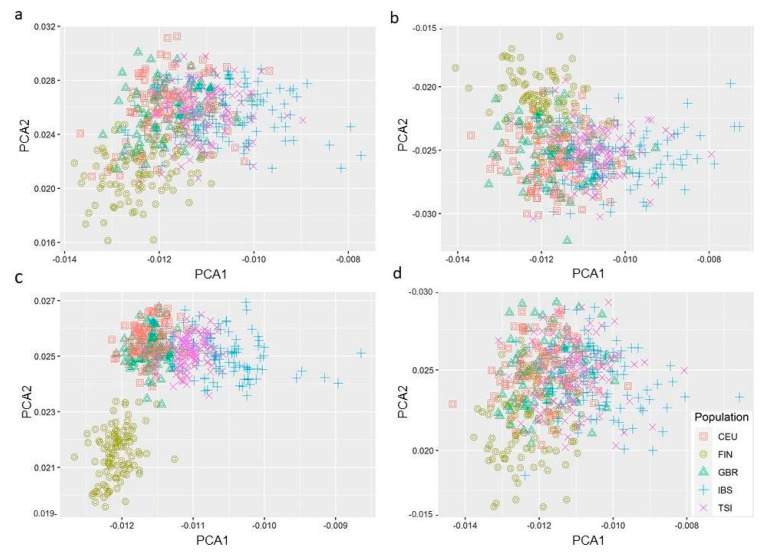
Principal component analysis from five European ancestries for the following canonical signalling pathways: (**a**) Caveolar-mediated Endocytosis (**b**) MS*P-*RON Signalling (**c**) Hepatic Fibrosis/ Hepatic Stellate Cell Activation (**d**) Communication between Innate and Adaptive Immune cells. The populations include CEU: Utah residents with Northern and Western European Ancestry, FIN: Finnish population, GBR: Great Britain (England and Scotland), IBS: Iberian (Iberian Population in Spain), TSI: Tuscany (Tuscany in Italy). Genes obtained from pathway analysis (IPA, Qiagen) and gene locations (GRCh37/hg19 built) included a ± 1 Mb window. Genome data outsourced from 1000 G phase III v.5b. PC analysis. PC analysis was performed using PLINK software v1.9b.

The PC analysis results are depicted in Figure 3a–d. Out of the four pathways analysed, only the genetic variants related to the *Hepatic Fibrosis/HSC Activation* (Figure 3c) represented a genetic differentiation for some of the populations tested, with the Spanish/Italian populations clustering together and the Finnish population segregating independently. The divergent segregation of the latter could reflect its well-known unique genetic background [75,76]. However, the fact that it is only segregated in one of the four pathways gives us a degree of confidence that the results observed here harbour a potential clinical significance with the symptoms associated with COVID-19 severe disease.

Next, the 20 top signalling pathways associated with 21 genes initially reported in aggressive COVID-19 disease were overlapped with a cut-off of five common genes (Figure S6). Interestingly, the *Hepatic Fibrosis/HSC Activation* pathway was the only canonical pathway that shared common genes with the host’s *Coronavirus Pathogenesis signalling pathway*. These common genes, *CCL2, IL6, SERPINE1, IL1β* and *IFNAR1* are highlighted in purple in Figure S7. Briefly, it shows the nuclear downstream effects of cytoplasmatic SARS proteins (SARS 3A, SARS 3b and SARS 7A) interfere in the transcription of *IL6, CCL2 and IL1β* via the activation of transcription factors such as FOS-JUN and NF_k_B, leading to hypercytokinemia, tissue inflammation and fibrosis.

### 3.6. Functional SNPs within Genes Associated with Severe COVID-19

In order to identify functional SNPs responsible for genetic predisposition to aggressive COVID-19 disease, an in silico analysis was undertaken for SNPs (including in linkage disequilibrium) that showed significant MAF differences in Spanish/Italian populations (case sample set) versus the rest of Europe (control sample set) (Table S10). This analysis revealed 26 functional SNPs in genes associated with COVID-19 aggressiveness (Table 4). SNPs in *IL1B* and *TNF* presented the highest significant MAF differences between cases and controls (CHISQ = 37.73 and 36.98, respectively) and RegulomeDB score (0.6–0.7). Additionally, significant MAF differences between cases and controls also identified rs60075147 in *Interferon α and β receptor subunits 1 and 2* (*IFNAR1/IFNAR2*), which was the only SNP that scored the highest possible RegulomeDB score. Genes *SERPINE1* had six functional SNPs (rs75339477, rs79520712, rs62465617, rs62465619, rs62465620, rs376313468) and the *LDH* had three functional SNPs (rs56357050, rs10841699, rs2196017).

Our previous overlapping analysis between the 20 top pathways associated with aggressive disease identified *IFNAR1* and *SERPINE1* as common genes between the *Host-Coronavirus Pathogenesis* and the *Hepatic Fibrosis/ HSC Activation*. These combined with our MAF analysis and a regulatory function analysis suggest an important role of these genes and the SNPs in these genes in the Spanish/Italian populations’ severe responses to SARS-CoV-2.

## 4. Discussion

The viral SARS-CoV-1 and SARS-CoV-2 spike (S) receptor binds with the highest affinity to the human receptor angiotensin-converting enzyme II (ACE2) [12,13,14,18,24,45,46,47,50,51,52,53], assisting viral host recognition and cellular entry, with studies showing a greater affinity with the novel SARS-CoV-2 than its predecessor, SARS-CoV-1 [77]. After binding, the transmembrane protease serine 2 (TMPRSS2) is the most common S protein co-activator [13,46,48,49]. Several additional human transmembrane receptors and co-activators have also been identified in vitro and in silico [2,13,14,18,24,49,50,51,52,53,54,78,79,80], indicating SARS-like viral RNA entry into the host cell can occur through a different molecular mechanism. The novel SARS-CoV-2 specific furin-like cleavage site on its S protein has been associated with a higher pathogenicity [63,81].

To escape immune detection and/or suppression, SARS-like viruses interfere with the host’s protein translation [55,56] (including those associated with antiviral response) and hijack key immune response regulators such as interleukins (IL) and chemokines [10,57,58,59,60,61,62,82,83,84,85,86]. Additional strategies to replicate, assemble and release viral particles have also been reported [16,47,87]. The most common immune signalling pathways affected by SARS-like viruses are those regulated by interferons (IFN) and NF-κB [10,57,58,59].

Finally, a correlation between cytokine and IL imbalances with COVID-19 severity was reported at the beginning of the pandemic [10,20,63], with early lymphopenia (low blood lymphocyte counts) identified as early markers of disease severity and low survival [63,64,88,89,90].

Since the beginning of this study, an exponential growth of knowledge regarding SARS-CoV-2 and the disease it causes has been observed, with over 44K publications related to COVID-19 by April 2021 (pubmed.ncbi.nlm.nih.gov). However, during the first months of the pandemic, available clinical information regarding the first COVID-19 patients and the specific characteristics of SARS-CoV-2 were only starting. At that time, we consolidated a list of candidate genes to bind the S protein [12,13,14,18,24,45,46,47,50,51,52,53] or to prime it [13,46,48,49] in SARS-CoV-1, SARS-CoV-2 or other related coronaviruses. Additional candidate genes were included for their role in assisting viral immune evasion [10,57,58,59,60,61,62] and promoting an aggressive COVID-19 clinical phenotype [10,20,63,64,65].

In silico protein interaction analysis (physical and functional) with those 21 genes associated with severe COVID-19 disease showed that most of the respective encoded proteins act as links that crosstalk between functionally related clusters such as TNF, IL-1B, IL-2, IL-6, IL-10, IFNAR1/2 and VEGFA. Similarly, ClueGO analysis showed TNF, several interleukins and cytokines, as well as IFNG, actively interact through certain pathways. The pathways that showed greater interconnection were *Regulation of Chronic Inflammatory Response* (typically seen in severe patients in several tissues), *Negative Regulation of Natural Killer Cells*, which reduces the successful inhibition of microbial infections [91] and *Positive Regulation of Calcidiol 1-monooxygenase Activity*. *Calcidiol 1-monooxygenase* enzyme, also known as the *VDR* gene, regulates the active form of Vitamin D. Vitamin D is an active regulator of the immune response and Vitamin D deficiency has been associated with a more aggressive form of COVID-19 and poor prognosis [92].

Our signalling pathway analysis identified the *Caveolar-mediated Endocytosis* as the most significant cascade associated with all the candidate genes and the *Hepatic Fibrosis/HSC Activation* as the canonical pathway most significantly associated with COVID-19 severe disease, respectively. From the four PC analyses of the genetic variants located in the four pathways previously identified, only the *Hepatic Fibrosis/ HSC Activation* signalling showed segregation amongst the populations studied. The Spanish/Italian populations clustered together and away, while the Finnish did so independently. Nevertheless, these results have their own limitations. Additional tools, such as fastStructure (Raj et al., 2014) or Admixture (Alexander et al., 2009), can be used to validate if the SNPs in the genes with this pathway can identify the same population structure. 

Initially, we found it slightly surprising that *Hepatic Fibrosis/HSC Activation* was the most associated pathway to aggressive disease, but the liver and HSCs play an important immunological role to protect themselves from infections [93]. For instance, cytokines (TNF-α, TGF-β, IFN-γ), IL (IL6) and chemokines (CCL21) are released from the liver to activate HSCs and resolve liver injury (Figure S4a). When the external strain resolves, activated HSCs undergo apoptosis and become quiescent. But if the liver is under severe or repeated damage, such as during a viral infection, HSCs constitutively proliferate, leading to liver fibrosis [93,94,95] or becoming immunoreactive [96]. As an example, both HCV and HBV promote liver fibrosis by these very molecular mechanisms [96,97], with both viruses being the major cause of chronic liver disease [98].

Liver injury as a comorbidity in severe COVID-19 patients has been recently reported, further supporting our research outcome [99,100,101,102,103]. Liver tissue damage after SARS-CoV-2 infection has been reported as the second most common organ damage after the lung [104]. However, it is also conceivable that this incidence may be even higher. COVID-19 patients with no pre-existing liver conditions can present 22–71% higher levels of AST (aspartate aminotransferase) and alanine transaminase increase (ALT) [105], both of which are liver damage biomarkers. It is plausible, then, that in the case of the Italian population, with the highest European incidence of HBV/HCV [106], individuals previously infected by either of these two viruses are more prone to develop liver damage after a subsequent SARS-CoV-2 infection, leading to a more severe response.

Following the PC analysis results, and in the context of presenting the highest COVID-19 related deaths in Europe at the time of the study, we looked at data from Spanish/Italian studies with available hepatic clinical and pathological features. A retrospective study from Spain with hospitalised COVID-19 patients (N = 1393) showed only 1.3% of the total cohort had liver cirrhosis [107]. Interestingly, after analysing only those patients admitted to the Intensive Care Unit (ICU), we found that 97% (N = 575) of them had above-normal AST levels, compared to those not admitted to the ICU,, and patients < 65 years showed significantly higher AST levels (*p* < 0.001) [107]. ALT values were not significantly higher. A different Spanish study observed that while 4% (N = 48) of COVID-19 patients were reported to have a chronic liver disease at the time of hospital admission, 45% of the cohort that died from COVID-19 presented liver failure [108]. We then searched for clinical data from COVID-19 Italian patients. A study with over 480 COVID-19 patients reported liver disease and high levels of LDH (another common liver damage marker [109,110]) as the 6th and 7th clinical predictors of deaths in hospitals, respectively [111]. Despite this, only 1.7% of the patients were clinically classified with chronic liver disease at the time of admission. Finally, a retrospective study of COVID-19 in Lombardy (N = 3988), the first European epicentre of the pandemic, showed only 2.7% of the COVID-19 patients presented liver disease at hospitalisation [112], but almost half of the ones that presented it (47%) passed away later on.

Liver tissue damage as a direct consequence of SARS-CoV-2 infection may be partially explained by the high expression levels of *ACE2* in some liver-specific cells (hepatocyte and cholangiocytes), with some studies showing similar levels to those observed in alveolar T2 (AT2) lung cells, albeit fewer in number [17,49,113]. Also, a very high expression of two S protein co-activators, *furin* and *TMPRSS2*, have been reported in hepatocytes, with *furin* additionally showing high expression in macrophages and endothelial liver cells [113]. This could be one of the key molecular links that explain why the *Hepatic Fibrosis/HSC Activation* pathway scored the highest level of significance in severe COVID-19 patients.

As a note, clinical trials with COVID-19 vaccines either included a very small number of patients with liver disease (<0.6%) or were specifically excluded [114]. Since liver disease from different aetiologies are shown to be associated with immune reactivity or immune suppression [96], even in today’s mass immunisation context, our data suggest hospitals around the world, and perhaps particularly in Spain and Italy, should consider regular follow-ups of those past COVID-19 patients that presented high levels of liver damage markers (ALT, ASD or LHD) after vaccination.

To determine if specific genetic polymorphisms shared between Spanish/Italian populations were partially associated with severe disease, we performed a frequency analysis of functional SNPs in relevant loci. This analysis showed rs79750333 and rs2853982 in LD, with *IL1B* and *TNF* as the functional SNPs with the most significantly different MAF between Spanish/Italian and European populations. Six nearby risk variants have been identified by a GWAS in COVID-19 patients [115] (rs74209081, rs61339327, rs113970174, rs76925104, rs78033025, rs76422048—located with genome.ucsc.edu/ accessed on June 2020). Similarly, close to rs2853982, four COVID-19 SNPs have been reported in hospitalised COVID-19 patients (rs12206131, rs34441152, rs71563335, rs71563325—genome.ucsc.edu/). Interestingly, at the same time, our data pointed towards the risk variant rs79750333 (*IL1B*) having the most significant MAF difference between the populations of interest (*p* = 2.68 × 10^−10^). IL6-inhibitors in severe COVID-19 patients were being tested [116]. Recent data from several multi-national studies later showed these drugs have little effect in reducing COVID-19-related deaths and/or severe symptoms [117]. Instead, a recent clinical trial where an Italian cohort (N = 392) was treated with IL1 or IL6 inhibitors showed a reduction in the death risk when treated with the first one only (HR = 0.45, 95%CI 0.20–0.99, *p* = 0.047) [118], supporting the valuable output from our study.

Our second most significant SNP, rs2853982 (*p* = 4.59 × 10^−10^), points to *TNF*. The anti-TNF treatment results have been promising in multi-national clinical trials, with patients showing less COVID-19-related mortality [119] in several clinical studies done in Spanish/Italian patients with rheumatic diseases [120].

Next, we looked at the functional SNP rs60075147 (in the vicinity of *IFNAR1/IFNAR2*). As mentioned previously, interferons are mediators of the early antiviral signalling pathways and their receptors, therefore, play an important role in initiating the initial immune response. GWAS studies have identified rs13050728 and its closest gene, *IFNAR2*, to be in the five most significant risk variants associated with critically ill COVID-19 patients (*p* = 1.045 × 10^−16^, app.covid19hg.org/variants, accessed June 202o). This has also been reported in another GWAS, where the *IFNAR2* locus was associated with severe disease (rs2236757, OR = 1.3, 95%CI = 1.17–1.41, *p* < 0.00009); by increasing the levels of IFNAR2, the risk of developing severe disease was reduced (*p* = 0.0043) [121]. Furthermore, inherited autosomal deficiencies (both recessive and dominant) in *IFNAR1/2* have been identified in highly severe COVID-19 patients [122]. Although some clinical trials showed IFNβ1a treatment did not have any effect on mortality [123], additional considerations, such as time of treatment, IFN-isoform specific therapies or the patient’s genetic background, should be taken into account. For instance, co-treatment with currently used antivirals (lopinavir/ritonavir and/or chloroquine) and subcutaneous injections of IFNα2b was reported to decrease the in-patient days if offered earlier (25  ±  8.5 days vs 10  ±  2.9 days, *p*  =  0.001) [124], to delay the need of ICU admissions (*p* < 0.02), to increase survival (*p* < 0.0001) [125] and to provide a faster full recovery by day 15 (*p* < 0.05) [126].

While our study presents a workable model to identify key pathways and genes associated with a pandemic in its early days, there are limitations, including the limited access to genomic data from a broader variety of European populations, the lack of a validation cohort and the not-yet-available ancestry-specific germline data from COVID-19 patients. Recent GWAS in Spanish/Italian cohorts identified 3p21.31 and 9q34.2 susceptibility loci associated with aggressive COVID-19, both of which were not discovered in our study [127]. In addition, the genes list used in the current analysis is constantly updating and the analysis needs to be repeated to identify additional pathways associated with the severe disease. Further, functional validation of our identified pathways using in vitro and in vivo models will make a valuable contribution to the Covid research.

To conclude, our in silico multi-approach study carried out in the early stages of the current COVID-19 pandemic led to main findings and additional speculations. The *Hepatic Fibrosis/HSCs Activation* pathways play an important role in developing severe COVID-19 disease. SNPs in the chromosomal loci related to this pathway group together the Spanish/Italian populations away from the European countries and Finland, independently. Hospitalised patients from the first populations presenting minor alleles of rs79750333 and rs2853982 may ameliorate severe symptoms if offered either IL1β inhibitors or anti-TNF treatments, respectively, in their early stages. Also, Spanish/Italian hospitalised patients presenting rs60075147 may reduce disease severity after treatments to increase IFN-specific isoforms. Additional in vitro assays to elucidate cross-talk between SARS-CoV-2 and above-mentioned genes/proteins may assist in discerning one of the host’s molecular responses that lead to severe disease and provide potential prognostic biomarkers and/or therapeutic targets, with a special value in male patients for the latter.

## Figures and Tables

**Table 1:** Host cell candidate genes associated with the viral entry. Only those genes reported in more than one reference are depicted in this table. For the full list see Table S1.

Candidate Genes	Virus	Host Cell Response/Viral Mechanism	ASSAY Methods	Refs.
*ACE2*	SARS-CoV-1, SARS-CoV-2	High S binding affinity. Facilitating host cell recognition.	Multiple in vitro and in silico analysis.	[12,13,14,45,46,47]
*TMPRSS2*	SARS-CoV-1, SARS-CoV-2	S protein activator, leading to viral membrane conformational change and facilitating SARS virus.	Multiple in vitro and in silico analyses.	[13,46,48,49]
*BSG*	SARS-CoV-2, malaria, HIV, HepB and HHV	*Basigin* genes encode CD147 transmembrane glycoprotein recognised by several pathogens. CD147 directly binds to SARS-CoV-2 S protein affecting viral replication.	Review and in vitro. SARS-CoV-2 strain isolated from COVID-19 patients. Direct in vitro infection, Co-IP and ELISA.	[18,50]
*HAT*	SARS-CoV-1, HCoV (229E)	*Histone acetyltransferases* family, encoding for a family of cell nuclear enzymes. They contribute to SARS-CoV-1 entry, but are not essential for S protein activation.	In vitro. Both studies: gene cloning, lentiviral expression system, protein expression and cell-cell fusion analysis.	[46,48]
*CLEC4M*	SARS-CoV-1, EVD, Dengue, HCV, CMV, Sindbis, HIV	*C-type lectin domain family 4 member M* genes encode for L-SIGN membrane receptor, recognised by the S protein. Homozygous L-SIGN associated with SARS disease protective role.	In vitro. Infection of SARS-CoV-1 human cells, gene expression, cDNA library, IHC assays. Genetic risk association from SARS patients and controls.	[24,51,52,53]
*ANPEP, ENPEP, DPP4 (or CD26)*	ACE2 studies, HCoV-22944, MERS-CoV45	Closest co-expression of these three peptidases (R > 0.8) with ACE2 in different human tissues. HCoV-22944 binds to ENPEP while MERS to DPP4.	Single cell in silico ligand-receptor affinity assays. Data sourced from GEO, Human Cell Atlas, Viral Receptor and Membranome databases.	[2,49,54]
*Cathepsin-B-L*	SARS-CoV-1, SARS-CoV-2, MERS-CoV	Facilitates SARS-CoV-2 cell entry by virus–cell membrane fusion mechanisms but its inhibition does not disable virus entry.	In vitro. SARS-CoV-2 S protein pseudovirus system in the human lung cell model.	[13,14]

**Table 2:** Host cell candidate genes associated with viral immune system evasion. Only those genes reported in more than one reference are depicted in this table. For the full list see Table S1.

Candidate Genes	Virus	Host Cell Response/Viral Mechanism	Research Assay	Ref.
*40s*	Nsp1 studies	Encoding for ribosomal protein S3, interacts with viral Nsp1, inhibiting the host’s protein translation by capping the 5′mRNA.	In vitro. Reporter gene assays followed by transcriptomics, RNA immunoprecipitation and proteomics assays.	[55,56]
*CCL5, CCL3, CXCL10*	SARS-CoV-1,	These genes encode for I*P-*10 * protein. Increased levels in lung epithelial cells after Nsp1-direct activation of the NF-kB pathway. I*P-*10 showed specific u*P-*regulation in the COVID-19 lung model (when compared to SARS patients).	In vitro. Gene cloning, mRNA and protein expression analysis. Ex vivo. Lung tissue transfected with COVID-19 and gene expression analysis.	[10,57]
*STING1, TRAF3, TBK1,* IKKε	SARS-CoV-1, HCoV (NL63)	The SARS-CoV-1 PLP transmembrane protein interacts with STING, TRAF3, TBK1 and IKKε, disrupting the STING/TBK1/IKKε complex formation and suppressing the production of IFN-α and IFN-β, vital for initial innate immune response. PLP protein is highly conserved in both SARS-CoV viruses, highlighting the use of potential agonists for this protein as treatments.	In vitro. SARS-CoV-1 propagation, and plasmids expressing genes of interest’s co-transduction. Co-IP and ubiquitination signalling detection. In silico. Homology alignments of both SARS viruses, approved compounds database screening and homology models predictions.	[58,59]
*ADP-ribose*	ssRNA	After binding to Nsp3, post-translational modification of PARP15, PARP14 and PARP10 is associated with anti-viral response.	In vitro. Cloning, gene expression, mutagenesis, protein purification and crystallization. In silico, sequence alignments, glycosylation sites’ predictions and 3D mapping.	[60,61,62]

40s: 40 subunit, *CCL5*: C-C motif chemokine ligand 5, *CCL3*: C-C motif chemokine ligand 3, *CXCL10*: C-X-C Motif Chemokine Ligand 10, *STING1*: stimulator of interferon response cGAMP interactor 1, TRAF3: TNF Receptor Associated Factor 3, IKKε: inhibitor of nuclear factor kappa-B kinase subunit epsilon, Nsp1: non-structural protein 1, SARS: Severe acute respiratory syndrome, HCoV: human coronavirus, ssRNA: single strand RNA, * previously known as I*P-*10, now CXCL10: C-X-C Motif Chemokine Ligand 10, NF-kB: nuclear factor-kB, PLP: papain-like protein, COVID-19: coronavirus disease 2019, PARP genes: Poly(AD*P-*Ribose) Polymerase.

**Table 3:** Dysregulated genes reported in first COVID-19 hospitalised patients.

Candidate genes	Disease	Reported observations	Ref.
*IL1β, IFN-γ, CXCL10,* and *MCP-1*	SARS and COVID-19	Increased in SARS and COVID-19 patients. Associated with Th1 cell immunity aberrant response and ARDS	[20]
*IL4 and IL10*	COVID-19	Increased in COVID-19 patients. Associated with Th2 cell immunity response, facilitating further ARDS	[20]
*IL2R and IL6*	COVID-19	High expression levels positively correlated with the severity of the disease	[10,20,63]
*CXCL10, MCP-1 and TNF-α.*	COVID-19	COVID-19 ICU patients have increased serum levels of these genes when compared to non-ICU COVID-19 patients	[10,64]
*IL1β, IL6, cRP*	COVID-19	High levels of IL1β are associated with a poor prognosis. IL6 and cRP are potential early risk biomarkers	[63]
SERPINE1 *	COVID-19	High levels of this protein is associated with vascular inflammation and a higher risk of thrombosis.	[63]
*LDH-hsCRP-lymphocyte*	COVID-19	Very accurate (>90%) predictive mortality biomarker signature	[65]

IL: interleukin, IFN: interferon, MC*P-*1: monocyte chemoattractant protein 1, CXCL10: C-X-C Motif Chemokine Ligand 10, hsCRP: high-sensitivity C-reactive protein, LDH: lactic dehydrogenase, SARS: Severe acute respiratory syndrome, COVID-19: coronavirus disease 2019, Th: T helper type, ARDS: Acute respiratory distress syndrome, ICU: intensive unit care, * encodes PAI-I protein.

**Table 4:** Functional SNPs within genes associated with severe COVID-19 and significantly different MAF in Spanish/Italian populations when compared to other European populations.

LD Gene	SNP	Chromosome Position	*p*-Value	Odds Ratio	CHISQ^®^	Regulome DB Score
		(GRCh37/hg19 Built)				
*IL1B*	rs79750333	chr2:114515437–114515438	2.68 × 10^−10^	2.96	37.73	0.67
*TNF*	rs2853982	chr6:31378750–31378751	4.59 × 10^−10^	3.15	36.98	0.6
*IL6*	rs10237482	chr7:22475177–22475178	2.57 × 10^−8^	0.46	31.87	0.8
*LDHA, LDHC*	rs56357050	chr11:18785334–18785335	5.68 × 10^−8^	0.46	30.56	0.6
*CXCL10*	rs114493545	chr4:123766808–123766809	3.11 × 10^−7^	6.24	22.82	0.8
*IFNAR1*, *IFNAR2*	rs60075147	chr21:33660824–33660825	4.31 × 10^−7^	2.74	24.48	1
*LDHB*	rs10841699 rs2196017	chr12:21060248–21060249, chr12:21061314–21061315	1.51 × 10^−6^	0.39	24.77	0.59,0.72
*IL10*	rs7530746	chr1:206712542–206712543	6.92 × 10^−6^	1.82	20.72	0.6
*IFNG*	rs741347	chr12:68631372–68631373	9.37 × 10^−6^	0.42	20.33	0.98
*VEGFA*	rs9381273	chr6:43976267–43976268	9.75 × 10^−6^	2.29	19.08	0.6
*IL4*	rs2243268	chr5:132013962–132013963	1.19 × 10^−5^	0.47	19.83	0.6
*PLAUR*	rs2356437 rs7258485	chr19:44352664–44352665, chr19:44353241–44353242	1.46 × 10^−5^	2.03	18.65	0.6
*CRP*	rs3806187	chr1:159750628–159750629	2.14 × 10^−5^	0.48	18.69	0.7
*CCL2*	rs1431994	chr17:32771454–32771455	3.01 × 10^−5^	1.9	17.14	0.6
*APOA2*	rs17381453	chr1:160514986–160514987	3.05 × 10^−5^	1.85	17.55	0.6
*IL2*	rs11937337	chr4:122373527–122373528	3.99 × 10^−5^	0.32	17.73	0.6
*LDHD*	rs147230411	chr16:74390997–74390998	7.65 × 10^−5^	6.9	13.77	0.69
SERPINE1 *	rs75339477	chr7:101012169–101012190	2.17 × 10^−4^	0.6	13.91	0.59
	rs79520712					
	rs62465617					
	rs62465619					
	rs62465620					
	rs376313468					
*CDH1*	rs696587	chr16:68546471–68546472	5.78 × 10^−3^	0.67	7.841	0.6

* The six SNPs are in high LD within 23 nucleotide regions presenting similar *p-*value, OR and RegulomeDB Score. *p*-values from Fisher’s exact test. ^®^Basic allelic test chi-square (1df) resulted from association analysis using PLINK. LD: linkage disequilibrium.

## Data Availability

Data is available in the form of supplementary tables.

## Supplementary Materials

The following supporting information can be downloaded at: https://www.mdpi.com/article/10.3390/genes14010022/s1, Figure S1: PubMed search between the 31st of March and 25th of May 2020; Figure S2: Caveolar-mediated Endocytosis signalling pathway; Figure S3: MSP-RON signalling pathway; Figure S4: Hepatic fibrosis and HSCs pathway; Figure S5: Communication between Innate and Adaptive Immune Cells pathway; Figure S6: Overlap of those canonical pathways with common candidate genes; Figure S7: Coronavirus Pathogenesis signalling pathway; Table S1: title; Genes identified through literature search; Table S2: title. List of IPA reported genes derived from Table S1; Table S3: Genes reported to be associated with COVID-19 disease severity and analysed for pathway analysis. Table S4: List of IPA reported genes derived from Table S3; Table S5: COVID-19 mortality rate in the European Union, calculated as per 100,000 people of the population for every country; Table S6: Genomic location of the genes of Caveolar endocytosis pathway; Table S7: Genomic location of the genes of MSP-RON pathway; Table S8: Genomic location of the genes of Hepatic Fibrosis/HSC Activation pathway; Table S9: Genomic location of the genes of Communication between Innate and Adaptive Immune Cells Pathway.
