# Supplementary material for: Severe COVID-19 May Impact Hepatic Fibrosis /Hepatic Stellate Cells Activation as Indicated by a Pathway and Population Genetic Study
Source: Genes (Basel). 2022 Dec 22;14(1):22. doi: 10.3390/genes14010022 (PMC9858736; doi:10.3390/genes14010022)
Supplement: Supplementary file 1 [file genes-14-00022-s001.zip › genes-1613732-supplementary.pdf]

PubMed searched terms		Number publications
COVID-19	+ genes	28
	+ protein interaction	40
	+ protein receptor	85
SARS-CoV-2	+ gene	66
	+ interactome	4
	+ protein interaction	31
SARS-CoV	+ interactome	7
	+ nsp protein	30
	Total	291

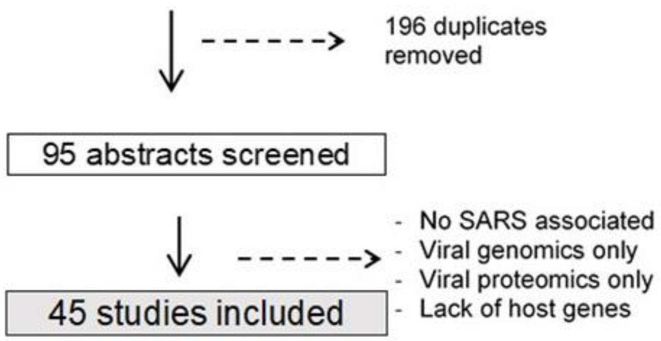

Supplementary Figure S1: PubMed search between the 31st of March and 25th of May 2020. Several searching terms were entered in the database to identify all reported SARS-CoV host interactome reported genes.

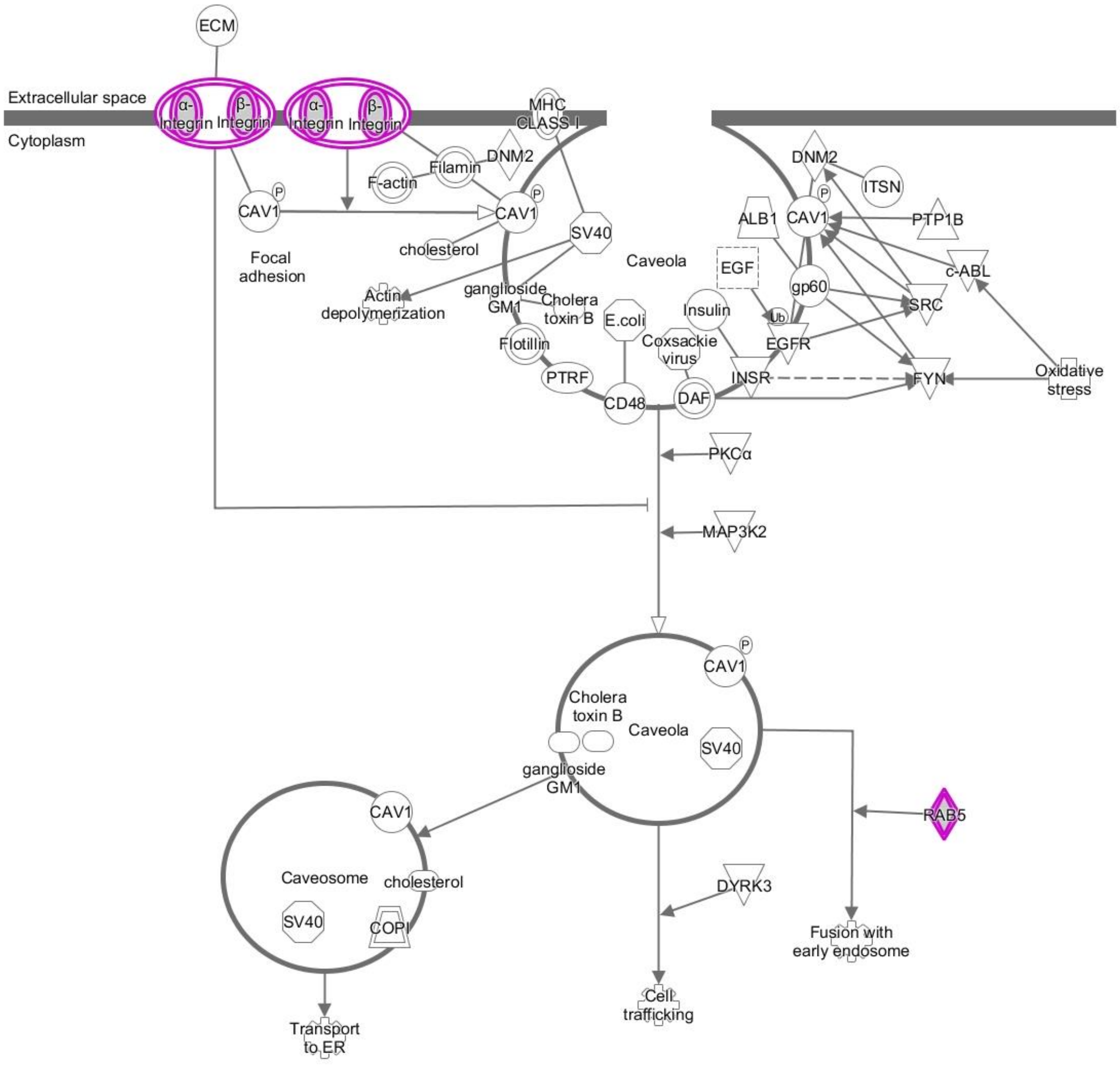

Supplementary Figure S2: *Caveolar-mediated Endocytosis* signalling pathway. Most significant canonical pathway associated with all candidate genes. Purple lines show the literature reported candidate genes in the pathway. Figure generated with IPA software.

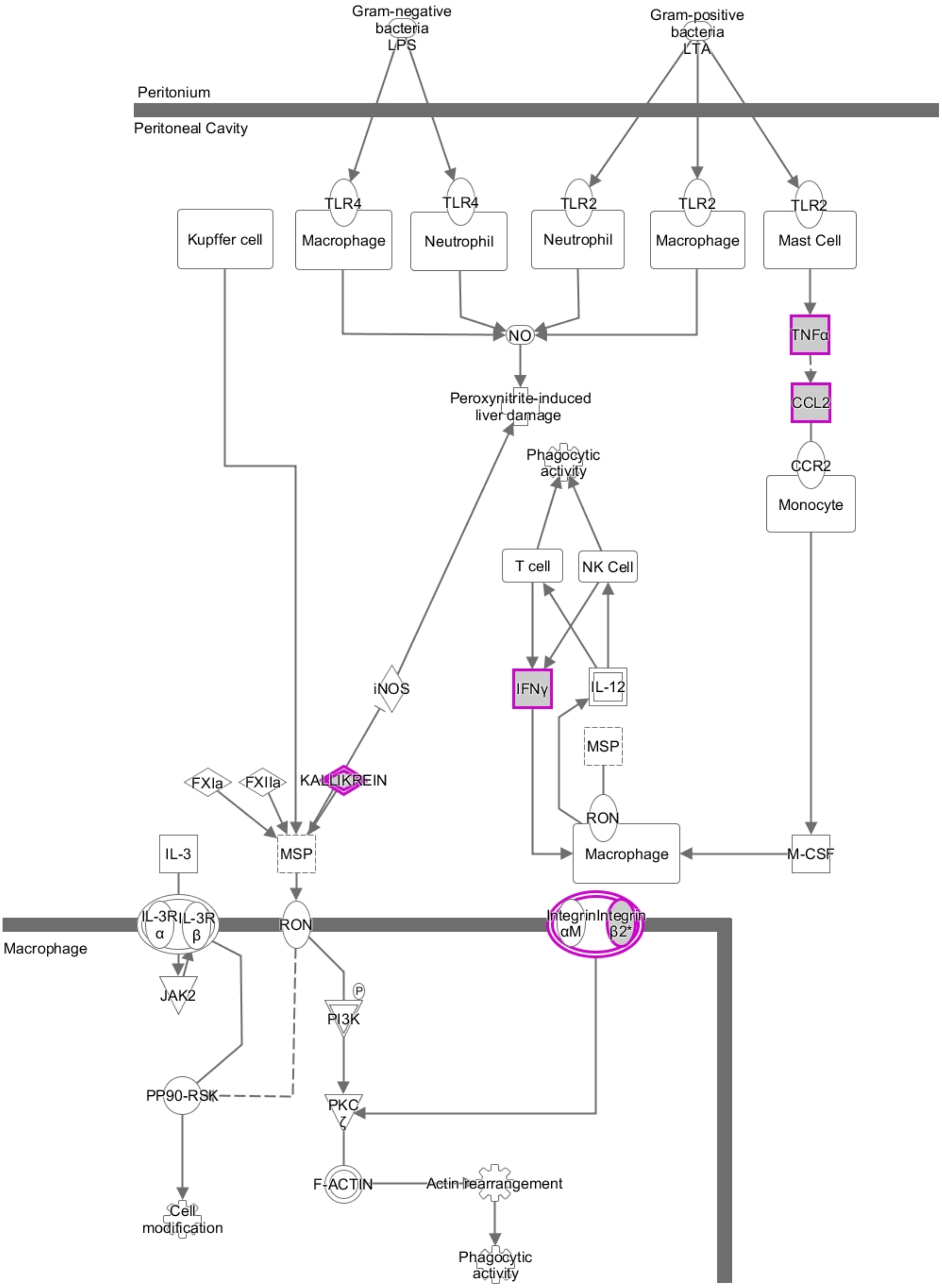

Supplementary Figure S3: ***MSP-RON* signalling pathway.** Second most significant canonical pathway associated with all candidate genes. Purple lines show the literature reported candidate genes in the pathway. Figure generated with IPA software.

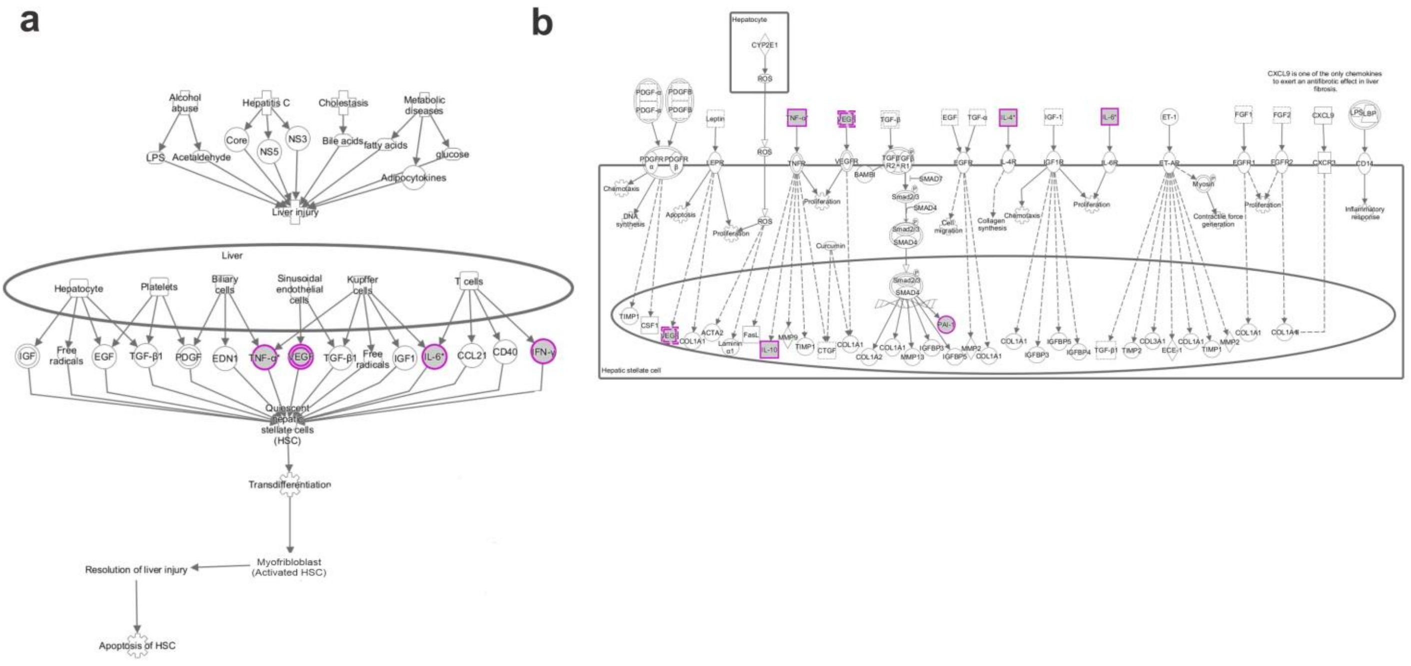

Supplementary Figure S4: ***Hepatic fibrosis and HSCs*** **pathway.** Second most significant canonical pathway associated with candidate genes identified with aggressive COVID-19 clinical outcome. Purple lines show the literature reported candidate genes in the pathway. Figure generated with IPA software.

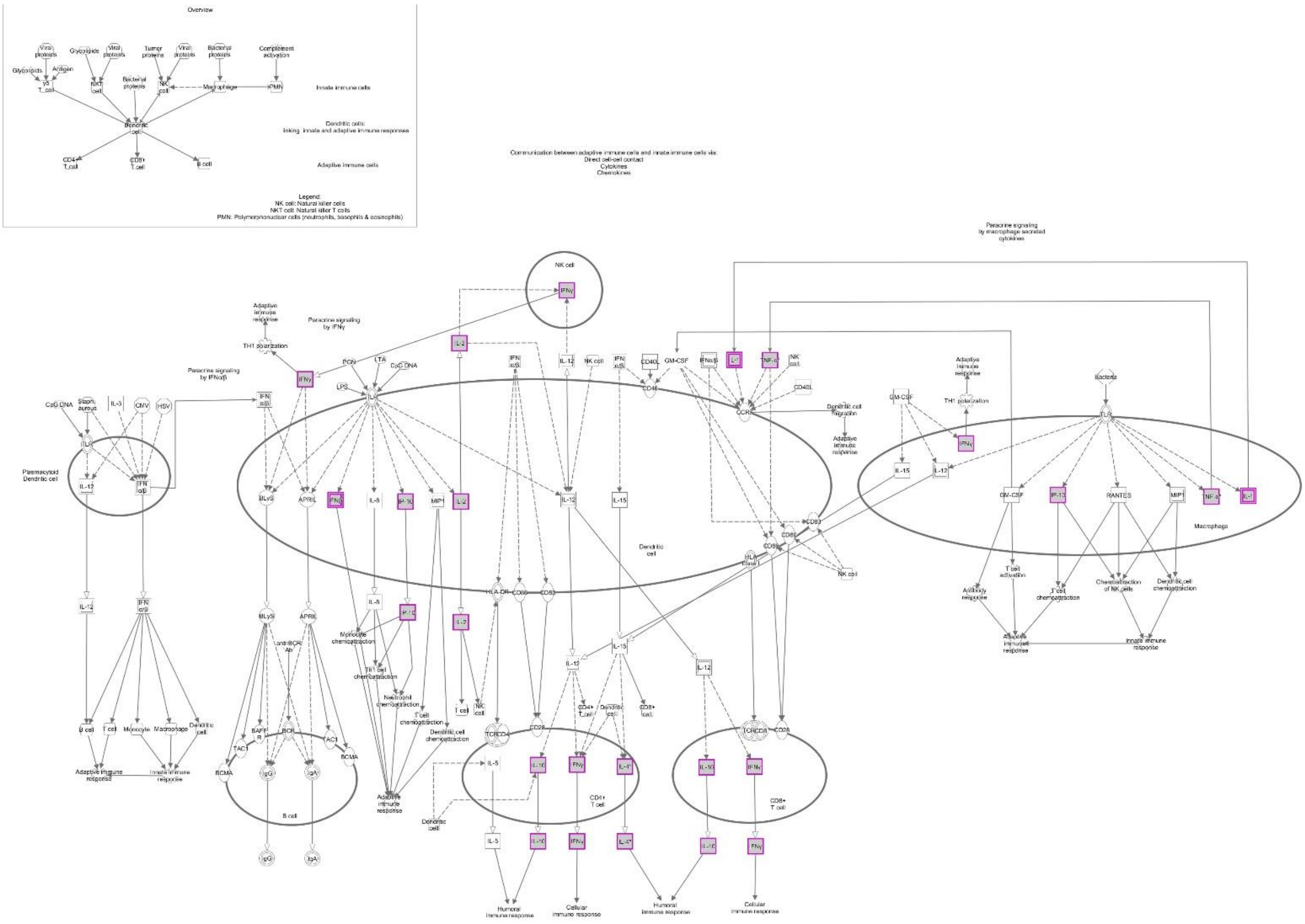

Supplementary Figure S5: ***Communication between Innate and Adaptive Immune Cells* pathway.** Second most significant canonical pathway associated with candidate genes identified with aggressive COVID-19 clinical outcome. Purple lines show the literature reported candidate genes in the pathway. Figure generated with IPA software.

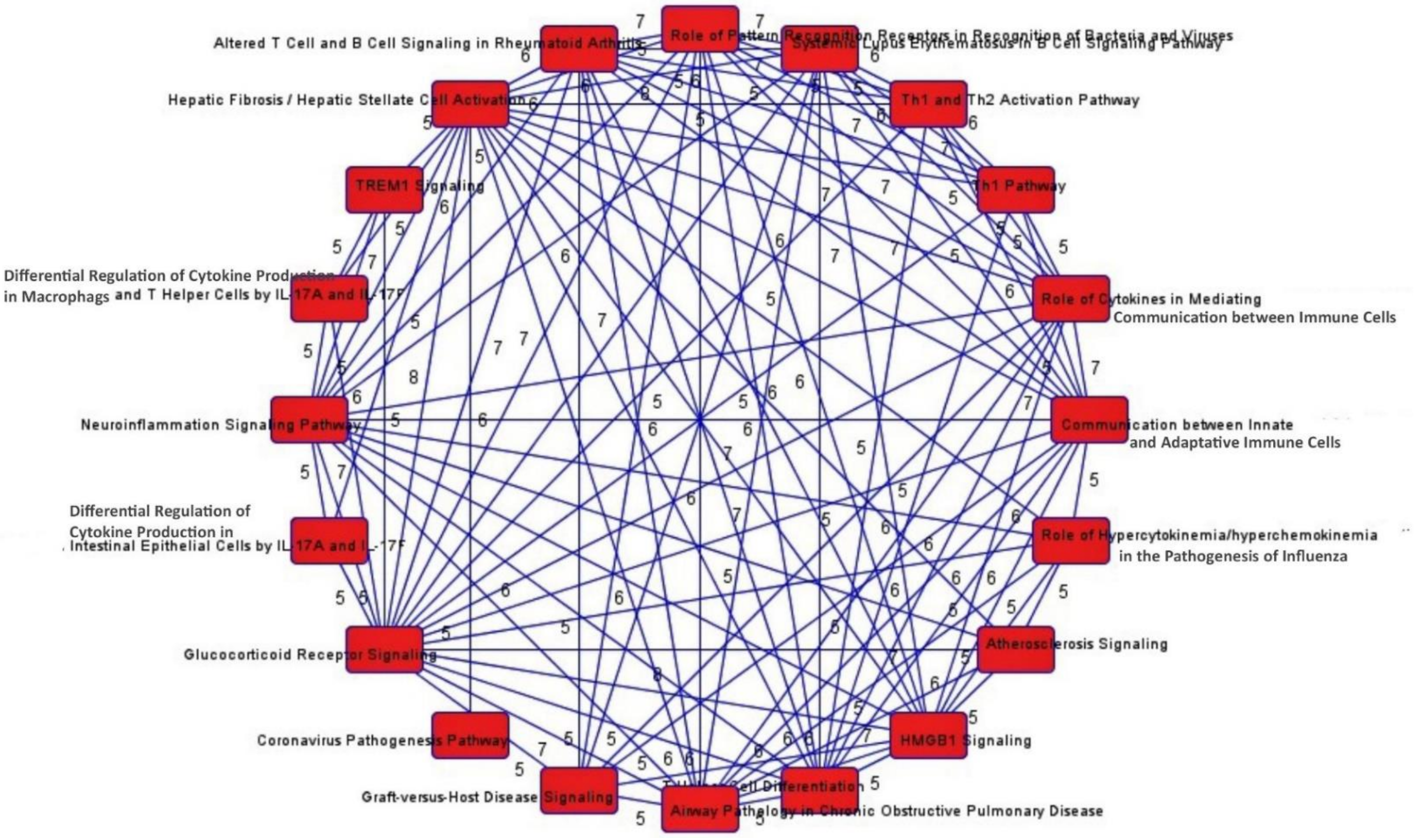

Supplementary Figure S6: **Overlapping of those canonical pathways with common candidate genes.** The 20 most significant pathways with a minimum of five common candidate genes associated with COVID-19 aggressive phenotype are shown. The bright red colour square reflects the high level of significance ($2.5\text{x}10^{-18} < P < 1\text{x}10^{-8}$). Figure generated by IPA generated software (Qiagen Inc.). P values calculated by Fisher's Exact Test.

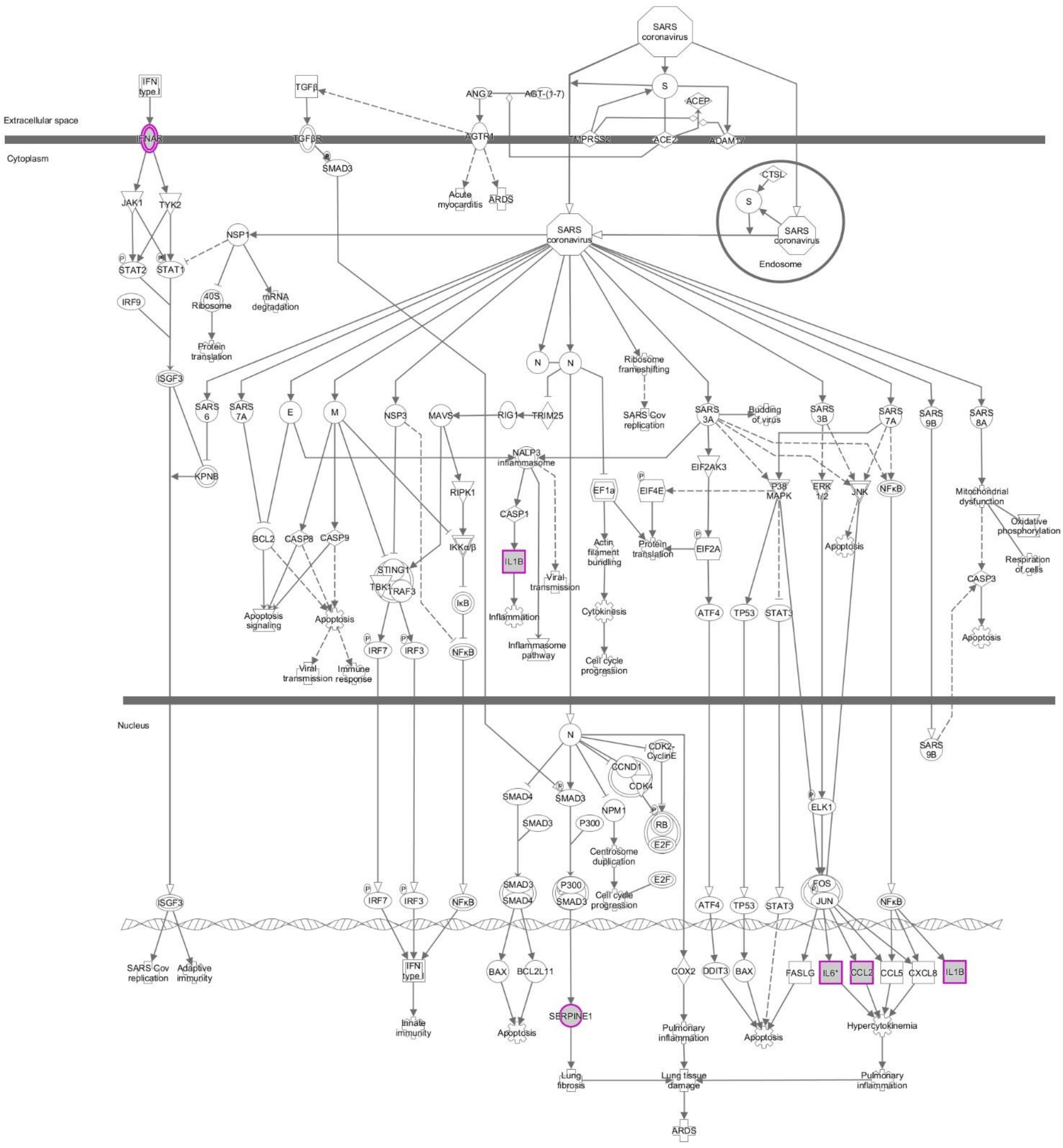

Supplementary Figure S7: ***Coronavirus Pathogenesis signalling*** **pathway.** Showing the host-coronavirus pathway. Purple lines show the location of the candidate genes associated with aggressive COVID-19 symptoms in the pathway. Figure generated with IPA software.
